# Significance of Hemorheological Tests During Mycoplasma Pulmonis Infection in Laboratory Rats

**DOI:** 10.3390/ani15040563

**Published:** 2025-02-14

**Authors:** Ádám Deák, Barbara Bedőcs-Baráth, Ádám Varga, Ádám Attila Mátrai, Tímea Bácskai, Krisztina Deák-Pocsai, Norbert Németh

**Affiliations:** 1 Department of Operative Techniques and Surgical Research, Faculty of Medicine, University of Debrecen, Moricz Zsigmond u. 22, H-4032 Debrecen, Hungary; barath.barbara@med.unideb.hu (B.B.-B.); varga.adam@med.unideb.hu (Á.V.); matrai.adam@med.unideb.hu (Á.A.M.); nemeth@med.unideb.hu (N.N.); 2 Doctoral School of Clinical Medicine, University of Debrecen, H-4032 Debrecen, Hungary; 3 Department of Anatomy, Histology and Embryology, Faculty of Medicine, University of Debrecen, Nagyerdei krt. 98., H-4032 Debrecen, Hungary; bacskai.timea@anat.med.unideb.hu; 4 Department of Physiology, Faculty of Medicine, University of Debrecen, Nagyerdei krt. 98., H-4032 Debrecen, Hungary; deak-pocsai.krisztina@med.unideb.hu

**Keywords:** hemorheology, *Mycoplasma pulmonis*, red blood cell aggregation, red blood cell deformability, sex differences, bacterial infection

## Abstract

*Mycoplasma pulmonis (M. pulmonis)* is a chronic infection that can appear in laboratory animal facilities. Deviations in hemorheological parameters during many pathological processes were detected, but it is unknown to what extent *M. pulmonis* infection causes changes in micro-rheological parameters. We performed our study on 25 symptomatic and asymptomatic Wistar rats, randomly selected from the entire population and presumably infected with *Mycoplasma*. From the blood samples, we determined the hematological parameters, red blood cell deformability under varying shear stress and varying osmolality, and the degree of red blood cell aggregation. We used the data of age- and sex-matched animals from our previous research as a control. Red blood cell count, hemoglobin, hematocrit values, and platelet count were significantly reduced in *Mycoplasma*-infected animals. Significantly reduced erythrocyte aggregation and deformability were detected. In conclusion, *M. pulmonis* infection causes significant changes in hemorheological and hematological parameters. Periodic measurement of these parameters can help monitor the infection.

## Simple Summary

Murine respiratory mycoplasmosis caused by the bacteria *Mycoplasma pulmonis* (*M. pulmonis*) is a common disease in wild and laboratory rodents. A natural *M. pulmonis* infection appeared in an experimental animal facility. Because it is currently unknown whether the infection causes changes in the hematological and hemorheological parameters and that these changes may indicate infection, we aimed to investigate those parameters in laboratory rats. We used the base values of similar-age male and female rats from our previous studies as a control. As a result, we observed several changes in hematological and hemorheological parameters and severe deterioration in the tissue and organs of ill animals. To our knowledge, we are the first to describe that *M. pulmonis* infection causes significant changes in those variables.

## 1. Introduction

Murine respiratory mycoplasmosis (MRM) or chronic respiratory disease (CRD) caused by *Mycoplasma pulmonis (M. pulmonis)* is one of the most common diseases in wild and pet rat populations. Among the laboratory animals, rats and mice are the primary hosts, but guinea pigs, hamsters, and rabbits are also susceptible [1,2,3,4,5,6].

The *M. pulmonis* infection is asymptomatic for a long time in rodents, but later it can appear with numerous symptoms [2,4,7]. The general symptoms include weight loss, hunched posture, ruffled coat, inactivity, and eye rubbing. Porphyrin staining around the eye (chromodacryorrhea) and nose can be observed. Respiratory symptoms are snuffing, sneezing, nasal discharge, tachypnea, and dyspnea. As a result of the involvement of the middle or inner ear, otitis media or interna can occur, which can cause torticollis [2,4,7,8,9]. *M. pulmonis* also affects reproductive organs. It can cause endometritis, perioophoritis, placental lesions in females, and testicular and spermatocyte degeneration in males [1,2,4,7,10,11]. The infection affects the immune system, reducing the delayed hypersensitivity response resulting in immunologically incompetent animals [12,13].

Significant changes in hemorheological parameters have been detected in human clinical cases and experimental animal models. Increased red blood cell aggregation in tumors and hematological disorders, such as β-thalassemia, were reported [14,15,16,17]. Congenital and acquired changes and diseases are associated with a significant deterioration of erythrocyte deformability. Reduced deformability is found in hereditary or acquired spherocytosis, elliptocytosis, stomatocytosis, sickle cell anemia, and iron deficiency states [18,19,20,21]. Deviations in hemorheological variables in animal models of diabetes mellitus, obesity, cardiovascular diseases, and sepsis were described [22,23,24,25]. 

Despite the fact that *M. pulmonis* can interfere with a wide range of experimental studies, there are no articles in the literature about the effect of infection on micro-rheological parameters. Therefore, we aimed to investigate hematological and hemorheological parameters in *Mycoplasma*-infected laboratory male and female Wistar rats.

## 2. Materials and Methods

### 2.1. Animals and Blood Sampling Method

The study was carried out according to EU regulations (EU Directive 63/2010) and the Hungarian Animal Protection Act (Law XXVIII/1998) regarding animal experimentation. The study was registered by the University of Debrecen Committee of Animal Welfare (permission registration No.: 24/2016/UDCAW). The animals were housed in a conventional animal facility, with a 12-h light/dark cycle, at a temperature range between 20–24 °C. Standard rodent nutrition pellets and tap water were available ad libitum.

Our study was performed on 25 (17 females, 8 males) symptomatic and asymptomatic Wistar rats, randomly selected from the entire herd. To confirm the infection, after the blood sampling, the whole carcasses of 5 (2 female, 3 male) animals, as well as formalin-fixed lungs from another 5 (female) animals, were sent to the Pathological and Bacteriological Laboratory of Debrecen, of the Veterinary Diagnostic Directorate of the Hungarian National Food Chain Safety Office (NFCSO). The carcass of one female rat was sent to GVG Diagnostics GmbH (Leipzig, Germany).

In NFCSO, PCR amplification was performed with 10 μL of nucleic acid in a 100 μL reaction mixture (50 mM KCI (Sigma-Aldrich Inc., St. Louis, MO, USA), 10 mM Tris-HCI pH 9.0 (Sigma-Aldrich Inc., St. Louis, MO, USA), 1.5 mM MgCl_2_ (Sigma-Aldrich Inc., St. Louis, MO, USA), 0.01% gelatin (Sigma-Aldrich Inc., St. Louis, MO, USA), 0.1% Triton X-100 (Sigma-Aldrich Inc., St. Louis, MO, USA)), 50 pmol of the primers, 0.2 mM each deoxynucleotide triphosphate (Merck KGaA, Darmstadt, Germany), and 0.2 U of Super Taq polymerase (Sigma-Aldrich Inc., St. Louis, MO, USA) based on van Kuppeveld et al. (1994) [26]. The thermal profile consisted of 40 cycles of denaturation at 94 °C for 1 min, primer annealing at 55 °C for 1 min, and primer extension at 72 °C for 2 min. The PCR test for confirming *M. pulmonis* infection included the following primers: forward primer GPO-3 (5′-GGGAGCAAACAGGATrAGATACCCT-3’) and reverse primer MGSO (5’-TGCACCATCTGTCACTCTGTTAACCTC-3’) [26]. The PCR product was sent to the Biotechnology Research Institute (Gödöllő, Hungary) for sequencing. Sequence data were analyzed using BLAST+ 2.11.0 (Basic Local Alignment Search Tool) software.

After the infection was confirmed by PCR, nucleotide sequence determination, and ELISA (GVG Diagnostics GmbH, Leipzig, Germany), 15 more animals were involved in this study.

The rats were anesthetized intraperitoneally (100 mg/kg, ketamine hydrochloride 10%; CP-Ketamin, Produlab Pharma BV, Raamsdonksveer, The Netherlands; 10 mg/kg xylazine-hydrochloride, 2%; CP-Xylazin, Produlab Pharma BV, Raamsdonksveer, The Netherlands), and blood samples were taken from the posterior vena cava into blood collection tubes containing K3-EDTA (BD Vacutainer^®^ Becton Dickinson, and Company, Franklin Lakes, NJ, USA). After blood sampling, the animals were euthanized intravenously (300 mg/kg, ketamine hydrochloride 10%; 30 mg/kg, xylazine-hydrochloride, 2%). The lung and uterus were removed for pathological examinations.

As a control (n = 22), we used the base values of similar-age, healthy, male (n = 8), and female (n = 14) Wistar rats used in our previous studies.

### 2.2. Hematological and Hemorheological Measurements

We determined the quantitative hematological parameters (Sysmex K-4500, TOA Medical Electronics Co., Ltd., Kobe, Japan). This instrument measures the white blood cell count (WBC), red blood cell count (RBC), and platelet count (Plt) parameters with the aperture-impedance principle, and the amount of hemoglobin (Hb) spectrophotometrically. The hematocrit (Hct), mean corpuscular volume (MCV), mean corpuscular hemoglobin (MCH), and mean corpuscular hemoglobin concentration (MCHC) are calculated parameters.

We measured the red blood cell deformability, which means the passive stretching of red blood cells under force, under varying shear stress (0.3–30 Pa), and varying osmolality (50–500 mOsm/kg) using a high viscosity medium (PVP solution, osmolality: 302 mOsm/kg, viscosity: 30.4–31.6 mPas), with the LoRRca MaxSis Osmoscan ektacytometer (Mechatronics BV, Zwaag, The Netherlands).

During the deformability measurement with variable shear stress, 10 µL of anticoagulated whole blood is suspended in 2 mL of PVP solution, and the device records the degree of elongation caused by the rising shear stress in the form of a diffraction image. Based on this, calculate the elongation index (EI) value describing the deformability. Additional parameters can be determined by parametrizing the obtained curves, such as the maximal EI (EI_max_) and the shear stress corresponding to half of it (SS ½).

For measurements with varying osmolality, 250 µL of anticoagulated whole blood is suspended in 5 mL of PVP solution. During the measurement, the device dilutes and concentrates the samples with one low and one high osmolality PVP solution, while the applied shear stress remains constant (30 Pa). It also calculates the EI values based on the recorded diffraction patterns. The device calculates the EI measured in a hypo-osmolal medium (EI min), the maximum EI at the peak of the curve (EI max), the EI value measured in a hyper-osmolal medium (EI hyper), thereby providing the corresponding osmolality values (Omin, O(EImax), O hyper) and the area under the curve.

The degree of red blood cell aggregation, which is the reversible binding of red blood cells in stasis or at a low shear rate, was determined by the Myrenne MA-1 erythrocyte aggregometer (Myrenne GmBH, Roetgen, Germany). Using 20 uL of anticoagulated whole blood, the device, following a disaggregation step (600 s^−1^ shear stress), determines the intensity of the transmitted light in the 5th and 10th seconds of the aggregation process in stasis or at the low shear rate, based on the light transmission principle. It calculates the aggregation index parameters from the measured intensity values (stasis: M 5 s, M 10 s; low shear rate: M1 5 s, M1 10 s).

### 2.3. Histological Examination

After formalin fixation (10% neutral formalin, Leica Biosystems Inc., Leider Lane Buffalo Grove, IL, USA) and paraffin embedding (Blue Ribbon paraffin, Leica Byosytems Inc., Deer Park, IL, USA), 4 µm sections were prepared from the lung samples and stained with hematoxylin-eosin (Sigma-Aldrich, St. Louis, MO, USA).

### 2.4. Statistical Analysis

For statistical analysis, paired t-test, Wilcoxon test, Student’s *t*-test, and Mann–Whitney rank-sum test were used, depending on data distribution, with SigmaStat Software 3.1.1.0 (Systat Software Inc., San Jose, CA, USA) software. Probabilities less than *p* < 0.05 are considered significant.

## 3. Results

### 3.1. Clinical Signs

In 96% of the examined rats, we observed porphyrinic discoloration around the eyes and all over the body, and most of them had ruffled coats. Sneezing, snuffing, and heavy breathing were observed in 36% of the infected animals. We did not observe signs of middle or inner ear inflammation (torticollis, ear discharge) in any of the animals.

### 3.2. Polymerase Chain Reaction (PCR) and Nucleotide Sequence Determination

Of the five carcasses sent for examination, the NFCSO showed positive results in four animals during the PCR tests. As a result of nucleotide sequence determination, all four PCR-positive animals were found to be infected with *M. pulmonis*.

### 3.3. Hematological Parameters

**Figure 1:**
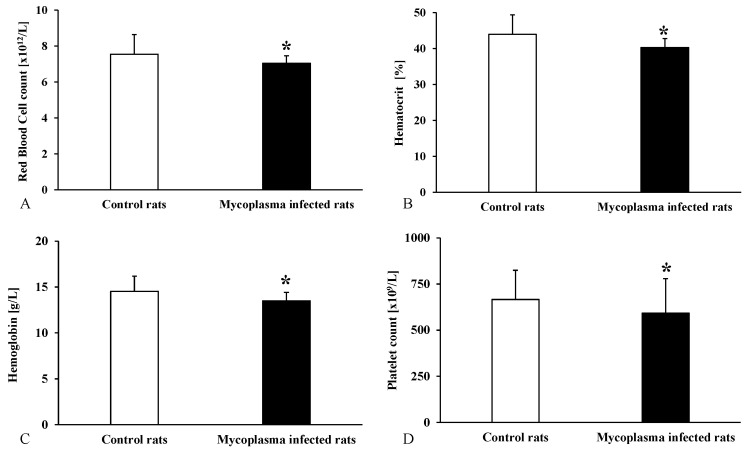
Red blood cell count (**A**), hematocrit (**B**), hemoglobin (**C**), and platelet count (**D**) values for all animals compared to the control group; Mean ± SD * *p* < 0.05 vs. Control (n = 22) (*Mycoplasma* infected n = 25).

Examining the hematological parameters, we found a significant decrease in the red blood cell (*p* < 0.001), hemoglobin (*p* < 0.001), and hematocrit (*p* < 0.001) values, as well as platelet count (*p* = 0.04) in the *M. pulmonis* group, compared to the control rats (Figure 1).

**Figure 2:**
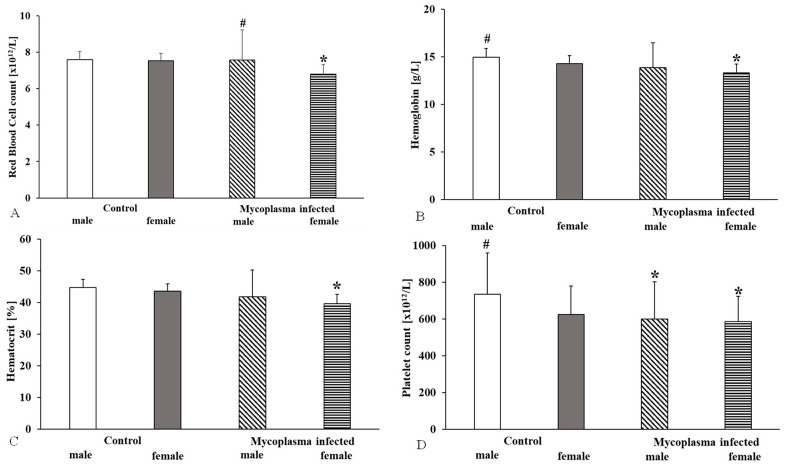
Red blood cell count (**A**), hemoglobin (**B**), hematocrit (**C**), and platelet count (**D**) values in a sex comparison, Mean ± SD * vs. Control male (n = 8); # vs. Control female (n = 14); *Mycoplasma* infected female (n = 17), (*Mycoplasma* infected male n = 8).

Comparing the male and female rats’ data, the red blood cell count was significantly higher in males with *M. pulmonis* than in females (*p* = 0.049); no significant difference was found compared to control males. In the case of females, we observed a significant decrease compared to the controls (*p* < 0.001). We found significant changes in hemoglobin (*p* < 0.001) and hematocrit (*p* < 0.001) values only in the case of female rats. In the case of the platelet count, we found a significant decrease in both sexes (males: *p* = 0.033; females: *p* = 0.011) (Figure 2).

**Figure 3:**
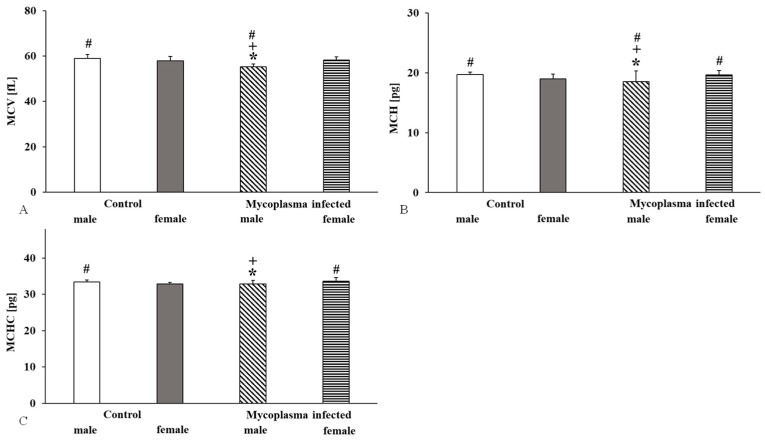
Mean cell volume (MCV) (**A**), Mean cell hemoglobin (MCH) (**B**), and Mean cell hemoglobin concentration (MCHC) (**C**) values in a sex comparison; Mean ± SD * vs. Control male (n = 8); # vs. Control female (n = 14); + vs. *Mycoplasma* infected female (n = 17), (*Mycoplasma* infected male n = 8).

In *Mycoplasma* infected males, the MCV (vs. control male *p* < 0.001; vs. control and infected females *p* < 0.001) and the MCH (vs. control male *p* < 0.001; vs. control females *p* = 0.012; vs. infected females *p* < 0.001) values were significantly reduced, while in females, the MCH (*p* = 0.003) was significantly increased compared to the control female group. Significant changes in the MCHC values were seen for infected animals. In infected males, we observed a decrease versus the control males and the infected females (vs. control males *p* = 0.044; vs. *Mycoplasma* infected females *p* = 0.007) and an increase in *Mycoplasma* infected females compared to control females (*p* < 0.001). Also, there was a significant difference between the control animals by sex: the MCHC value was higher in control males than females (*p* < 0.001) (Figure 3).

### 3.4. Hemorheological Parameters

**Figure 4:**
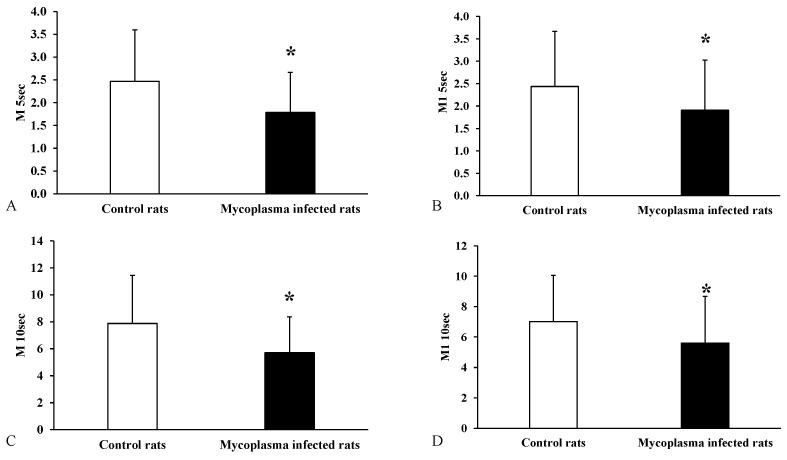
Aggregation index values for all animals compared to the control group, (**A**): M5 s; (**B**): M1 5 s; (**C**): M10 s; (**D**): M1 10 s; Mean ± SD * *p* < 0.05 vs. Control (n = 22) (*Mycoplasma* infected n = 25).

A significant decrease in the aggregation index values was detected in the group that was naturally infected with *M. pulmonis* (M5 s: *p* < 0.001; M1 5 s: *p* = 0.003; M10 s: *p* < 0.001; M1 10 s: *p* = 0.001) (Figure 4).

**Figure 5:**
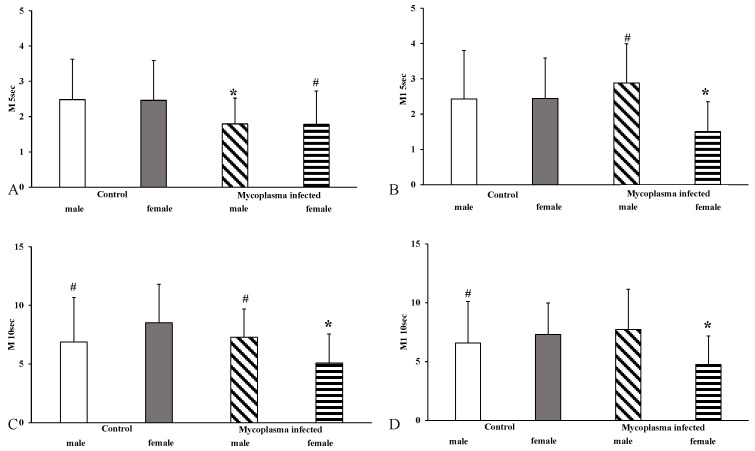
Aggregation index values in the sex comparison, (**A**): M5 s; (**B**): M1 5 s; (**C**): M10 s; (**D**): M1 10 s; Mean ± SD * vs. Control male (n = 8); # vs. Control female (n = 14); *Mycoplasma* infected female (n = 17), (*Mycoplasma* infected male n = 8).

In males, we found a significant increase in all parameters except the M5 s index parameter compared to the females with *Mycoplasma* (M1 5 s: *p* < 0.001; M10 s: *p* < 0.001; M1 10 s: *p* < 0.001), but there was no significant difference compared to the control group. In females, all index parameters showed a significant decrease compared to the controls (M5 s: *p* < 0.001; M1 5 s: *p* < 0.001; M10 s: *p* < 0.001; M1 10 s: *p* < 0.001) (Figure 5).

**Figure 6:**
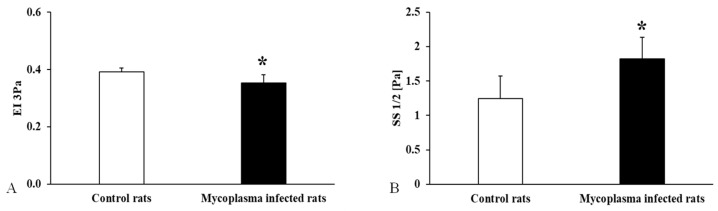
Deformability measurement data for all animals compared to the control group, (**A**): EI 3 Pa; (**B**): SS1/2; Mean ± SD * *p* < 0.05 vs. Control (n = 22) (*Mycoplasma* infected n = 25).

Examining the deformability measurement results, in the case of EI 3Pa, a significant decrease was visible in the *Mycoplasma* infected group (<0.001), and a significant increase was found in the SS1/2 values (*p* < 0.001) (Figure 6).

**Figure 7:**
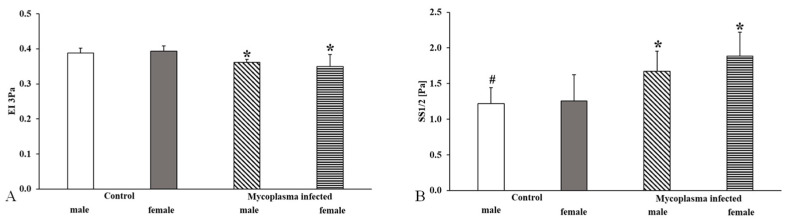
Deformability measurement data in the sex comparison, (**A**): EI 3 Pa; (**B**): SS1/2; Mean ± SD * vs. Control male (n = 8); # vs. Control female (n = 14); *Mycoplasma* infected female (n = 17), (*Mycoplasma* infected male n = 8).

We observed the same in the studies between the sexes (EI 3Pa: male vs. control male *p* < 0.001; female vs. control female *p* < 0.001; SS1/2: male vs. control male *p* < 0.001, vs. *Mycoplasma* infected female *p* = 0.033; female vs. control female *p* < 0.001) (Figure 7).

**Figure 8:**
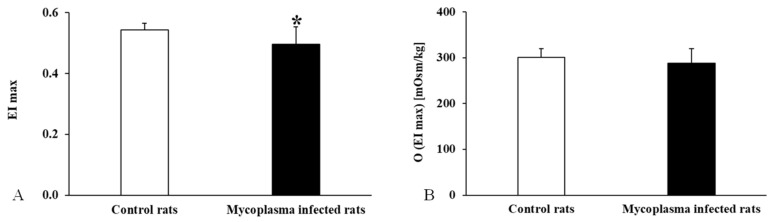
Osmotic gradient deformability measurement data for all animals compared to the control group, (**A**): EImax; (**B**): O(EImax); Mean ± SD * *p* < 0.05 vs. Control (n = 22) (*Mycoplasma* infected n = 25).

During the osmotic gradient deformability measurement, we found that the maximum elongation index was significantly lower in the infected group (*p* < 0.001), while no difference was found in the osmolality values associated with this elongation index (Figure 8).

**Figure 9:**
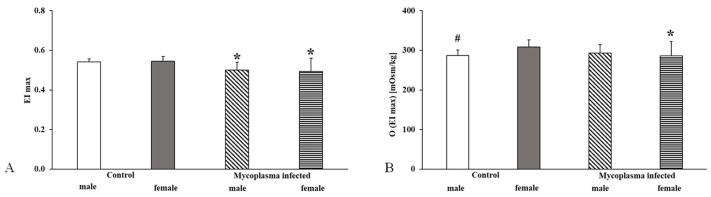
Osmotic gradient deformability measurement data in a sex comparison, (**A**): EImax; (**B**): O(EImax); Mean ± SD * vs. Control male (n = 8); # vs. Control female (n = 14); *Mycoplasma* infected female (n = 17), (*Mycoplasma* infected male n = 8).

Examining these parameters between the sexes, we saw that in the case of females, there was also a significant change in the osmolality values associated with the maximum elongation index (EImax: *Mycoplasma* male vs. control male *p* = 0.021; *Mycoplasma* female vs. control female *p* < 0.001; O(EImax): *Mycoplasma* female vs. control female *p* = 0.04), while we did not see a significant difference in males (Figure 9).

### 3.5. Macroscopic Observations

**Figure 10:**
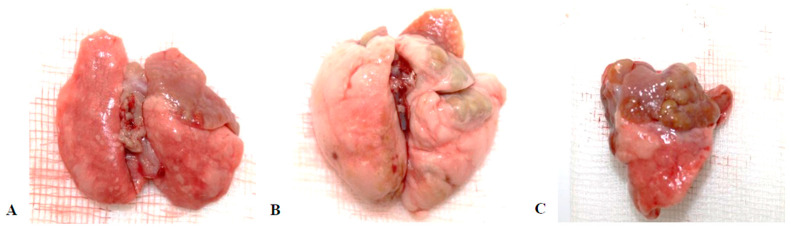
Macroscopic pictures of lungs, (**A**): Abscesses and grey hepatization; (**B**): Lung with abscesses and grey hepatization; (**C**): Lung with cobblestone lesions and red hepatization.

**Figure 11:**
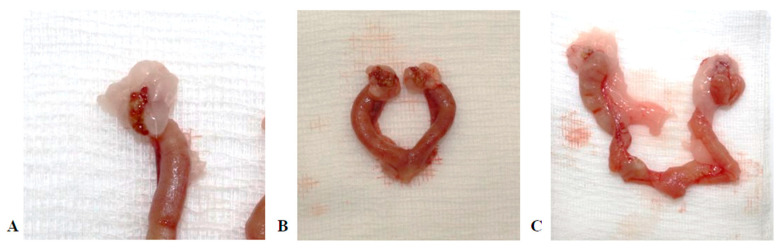
Macroscopic pictures of female rat reproductive tract. (**A**): Perioophoritis, salpingitis; (**B**): Suppurative endometritis; (**C**): Pyometra.

In several animals, suppurative inflammation, abscesses, grey and red hepatization, and cobblestone lesions were observed in the lungs (Figure 10). Perioophoritis, salpingitis, suppurative endometritis, and pyometra were diagnosed in female rats (Figure 11).

### 3.6. Histopathological Results

**Figure 12:**
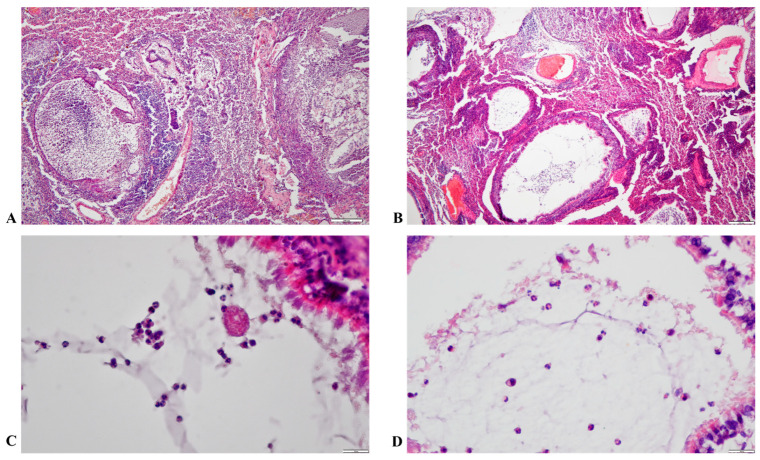
Microscopic pictures of the lung tissue (**A**,**B**): Lung tissue with abscesses, infiltration of inflammatory cells: lymphocytes and neutrophil cells (4×) scale bar = 200 µm; (**C**,**D**): Tissue disintegration in rat lungs, infiltrated with: lymphocytes, neutrophils and macrophages (40×) scale bar = 50 µm. Hematoxylin-eosin stain.

Interstitial pneumonia with peribronchial and perivascular lymphoid hyperplasia was diagnosed in the lungs of *Mycoplasma*-infected animals. Interstitial bronchopneumonia has also been described in formalin-fixed lung samples (Figure 12).

## 4. Discussion

The most common pathogens causing respiratory diseases in laboratory rodent facilities are bacteria such as Mycoplasma species, *Streptococcus pneumoniae*, *Corynebacterium kutscheri*, *Pseudomonas aeruginosa*, *Pasteurella pneumotropica*, and *Bordetella bronchiseptica*, and viruses as Sendai virus and Sialodacryoadenitis virus [4].

*M. pulmonis* infection can occur in variable percentages in conventional animal facilities. In addition to the disease that develops as a result of the infection, mainly affecting the respiratory tract, external and behavioral signs also appear [1,2,27,28]. In our study, neglected fur and sneezing were the first suspicious signs. Animals are often additionally infected with other pathogens (*Escherichia coli, Actinomyces species*); as a result, inflammation of the middle or inner ear may develop [1,2,4].

Several methods can be used for diagnoses of *M. pulmonis*, such as different serological methods as Multiplexed Fluorometric ImmunoAssay (MFIA^TM^, Charles River Laboratories International, Wilmington, MA, USA), Indirect Fluorescent Antibody (IFA), Enzyme-Linked Immunosorbent Assay (ELISA), bacterial culture, histological examination, and polymerase chain reaction (PCR). Matrix-Assisted Laser Desorption/Ionization-Time-Of-Flight Mass Spectrometry (MALDI-TOF MS) can also be used to analyze the protein composition [2,3,4,7,29,30].

After diagnosis, in everyday veterinary practice, the disease is treated with various antibiotics. The use of a certain antibiotic is often insufficient, so veterinarians resort to using different antibiotic combinations [1,4]. The treatment is regularly supplemented with nebulization and applying anti-inflammatory and mucolytic drugs. Despite the long and complex treatment, the animals cannot be considered free from *M. pulmonis*; therefore, when the infection appears in laboratory animal facilities, the animals are not treated. The only reasonable measure to eliminate the infection is complete depopulation and disinfection. Sanitation of cages and equipment (feeders, water bottles, sipper tubes, stoppers) should be performed with hot water and detergents or disinfectants. Vaporized chlorine dioxide and hydrogen peroxide are effective compounds for room decontamination. After disinfecting the area and instruments, the rooms can be restocked with healthy animals. In the case of unique strains, rederivation is applicable [31].

The prevention of *M. pulmonis* infection in laboratory animals must be ensured through strict health surveillance. Experimental animals can only be purchased from reliable, well-known, qualified vendors. Rodents should always arrive with the most up-to-date health report, including the results of the *M. pulmonis* screening. It is suggested that the animal facilities follow the Federation of European Laboratory Animal Science Associations (FELASA) recommendations regarding the health monitoring of rodent colonies in breeding and experimental units. The laboratory animal caretakers and technicians should not keep rodents as pets or have secondary employment where they could come in contact with pets or wild rodents.

The susceptibility and severity of the host to disease depend on factors such as strain, age, sex, immune status, and concurrent infection with co-pathogens [32]. Their transmission can be horizontal (direct contact, aerosol) or vertical (in utero) [2,7,33]. They have poor survivability in the environment [4]. Most species are commensal or do not cause serious diseases. Previously, *Mycoplasma* species were considered host-specific; however, in the case of some species, cross-infection may occur. Ferreira et al. examined the transmission rate of *M. pulmonis* from rats to humans. Based on their results, 22.6% of direct contacts (laboratory animal technicians) and 10% of indirect contacts (administrative and laboratory personnel) showed a positive PCR result. Interestingly, 15.5% of all contacts (both direct and indirect) were PCR positive, while among non-contact personnel, 2.82% showed PCR positivity [3]. Similar tests were carried out by Piasecki et al., who searched for the answer as to whether, although it is known that humans can be carriers of *M. pulmonis*, the infection causes symptoms and how viable the pathogen is in the new carrier [5]. PCR positivity was shown by 24.42% of the 86 pet rat keepers, 76.32% of the 13 laboratory technicians, and 25% of the 32 veterinarians. Seropositivity, which can be an indicator of active infection, was shown by 59.09% of all individuals included [5].

The infection primarily affects the respiratory system, initially without symptoms, but, with advancing age, difficulty breathing, sneezing, and runny nose can appear [2,3,4,8]. Macroscopically visible lesions appear in the lungs, and multiple fused abscesses often develop, with tan-white discharge. The lungs are watery and swollen, and pulmonary congestion and purulent or necrotic pneumonia are common [1,2,8,34]. In the majority of examined animals, we have seen necrotic lesions and abscesses in the lungs. In addition, lung hyperplasia was observed in several animals. In histological examination, lymphocytes, neutrophils, macrophages, and fibrin were described in pulmonary tissue. Degenerated and viable neutrophils, necrotic cell debris, and fibrin accumulate in the trachea. Neurogenic inflammation in the mucosa of the respiratory tract was also described [1,4,9,34,35].

Romero-Rojas et al. described that *M. pulmonis* infection causes changes in the hematological parameters. Ten days after infection, significantly decreased red blood cell and platelet count and an elevated white blood cell count were found compared to the control animals [36]. We also found significantly lower red blood cell count (anemia), hemoglobin concentrations, hematocrit, and platelet counts (thrombocytopenia) in the infected rats.

Differences between sexes were described previously in several hemorheological parameters. Whole blood viscosity is higher in men than women, and the same has been observed in rats and dogs. In the case of humans and beagle dogs, the degree of aggregation is higher in males, while in the case of rats, it is higher in females. Regarding the red blood cell deformability, in human and rat samples, females had higher elongation indices, and males had higher elongation indices in dogs. We can also find sex differences in fibrinogen levels in rats, where females’ values are higher than males’ values.

Hematological parameters also show sex differences. In rats, the white blood cell count is higher in males, as are the red blood cell count and hematocrit. Mean corpuscular hemoglobin and mean corpuscular hemoglobin concentration values are higher in female rats than in male rats [37,38,39,40,41]. In our study, the males with *M. pulmonis* infection had higher values than females, but only the females showed significant differences from the controls. The hemoglobin and hematocrit values significantly differed only in females, while the platelet count decreased significantly in both sexes. The MCV and MCH values were lower in males, while the MCHC values were elevated in both sexes. Despite the increasing number of studies on the topic, the physiological mechanisms of hemorheological and hematological differences between males and females are not fully elucidated. Factors such as genetic background, metabolic rate, the presence or absence of sex hormones (e.g., estrogen, testosterone), and the physiological or pathophysiological changes in hormonal levels have a complex effect on hematopoiesis, endothelial function, and the neuroendocrine and immunological systems and this requires more investigation [37,38,39,40,41].

Bacterial infections can cause changes in the hemorheological parameters [42]. Czepiel et al. found increased aggregation and plasma viscosity in *Clostridium difficile* infection and described reduced deformability under a low shear rate and increased deformability under a high shear rate [43]. Several researchers have investigated rheological deviations in experimental sepsis models. Yeom et al. found elevated whole blood viscosity and red blood cell aggregation in a lipopolysaccharide (LPS) induced ex vivo sepsis rat model [44]. A reduced RBC deformability and increased aggregation during sepsis were observed [45,46,47]. In an *Escherichia coli*-induced sepsis porcine model, reduced microcirculation, platelet count, increased whole blood viscosity, and decreased plasma viscosity were found. In the same research, reduced deformability and slightly reduced aggregation were described. In the case of osmotic gradient deformability, increased EI min and O (EImax) and decreased O min and EI max were observed [25,48]. Silva et al., in their experimental study, induced a severe bacterial infection with *Salmonella enterica* in mice. A hyperactivation of platelets and eryptosis of RBCs in different phases was described [49]. Ben Ami et al. reported that aggregation correlates with C-reactive protein (CRP) and fibrinogen levels, and that the RBC aggregation increased during bacterial infection [50].

There may be several factors behind why hemorheological parameters deteriorate due to infection. During inflammatory processes, the amount of reactive oxygen species (ROS) increases, which reduces the activity of endothelial nitric oxide synthase (eNOS), thus reducing the amount of nitric oxide (NO), which causes endothelial dysfunction. ROS directly damage the cell membrane through lipid peroxidation, destroy transmembrane proteins by forming sulfhydryl bonds, and are also harmful to hemoglobin molecules and structural proteins [51]. ROS reduce red blood cell deformability and aggregation [52].

Infections are often accompanied by elevated body temperature, even fever. Grau et al. described that red blood cell deformability significantly decreases in the event of a COVID-19 infection, where fever is also a typical symptom, which cannot be offset by increased NOS activity. The red blood cells of the patients were permanently elongated, and protrusions appeared on their membranes. No significant change in aggregation was found, but it was observed that higher shear stress is required to maintain the aggregation-disaggregation balance [53]. In previous research, we investigated the effect of temperatures higher than physiological on red blood cell deformability, using blood samples set to 10% hematocrit in various species. As the temperature increased (40–43 °C), the deformability decreased significantly, and showed differences between species. The greatest deterioration was found in the human samples, followed by the pig, rat, and dog specimens [54]. It has been described that red blood cell deformability deteriorates in case of fever or febrile conditions [55,56,57]. It can be assumed that the increased temperature also harms the micro-rheological parameters of the red blood cells [55,57].

As a result of infections, patients are often dehydrated. As a consequence of dehydration, the fluidity of the plasma decreases, thus increasing its viscosity and the hematocrit and erythrocyte aggregation increase. The membrane of the cells becomes more rigid, therefore reducing the deformability of the red blood cells [58].

There is no article in the literature about the effect of *M. pulmonis* infection on hemorheological parameters. In our study, red blood cell aggregation was significantly reduced in the naturally infected group, and we also saw a significant difference between the sexes; the aggregation indices of infected males were higher than those of infected females. A significant deterioration of deformability was visible in the *Mycoplasma*-infected group in both sexes; however, only the SS1/2 parameter showed a significant difference between sexes. During the osmotic gradient deformability measurement, the maximum elongation index values in the *Mycoplasma*-infected group were significantly lower compared to the control. At the same time, no significant change was found in the corresponding osmolality values, although the reduced value is visible. In this case, we found no difference between sexes.

## 5. Conclusions

To our knowledge, we described, for the first time, that *M. pulmonis* infection causes significant changes in hematological parameters, which manifested in decreased red blood cell count and reduced hematocrit values, and hemorheological parameters, in the form of impaired deformability and reduced aggregation in laboratory rats. For the first time, we described the sex differences in the deterioration of the above-mentioned parameters caused by natural *M. pulmonis* infection. The periodic measurement of these parameters, as well as the knowledge of the changes we describe, can help in monitoring the infection.

## Data Availability

The raw data supporting the conclusions of this article will be made available by the authors, without undue reservation.
